# Teeth below Medium in Size.—Their Treatment

**Published:** 1894-03

**Authors:** Wm. W. Belcher

**Affiliations:** Seneca Falls, N. Y.


ARTICLE III.

### TEETH BELOW MEDIUM IN SIZE.—THEIR TREATMENT.

BY WM. W. BELCHER, D. D. S., SENECA FALLS, N. Y.

Read before the VIIth District Dental Society.

During our last census considerable discussion was had and indignation manifested by certain dentists as to whether or not they should be classed as manufacturers; whether or not dentistry was a mechanical or a professional calling. The making of a set of teeth is certainly a mechanical operation, likewise the burring out of a simple cavity and the filling thereof with amalgam or gold. But where a careful examination of the mouth is made to determine the best method of constructing a denture; where the trained eye is called into play to determine the most suitable tooth for the sex and temperament of the patient; in operative dentistry, where the operation necessitates not only the mechanical art of stopping a cavity, but also calls into play the judgment as to the most suitable filling material for the patient, the tooth, and its position in the dental arch, different cases requiring different treatment, then the mechanical ends and the professional begins. The treatment of teeth below medium in structure, I find, calls for more professional attention than mechanical.

What is the most suitable material for these teeth? Gold? How many times in the course of a year you examine mouths studded with soft, frail teeth and find patients completely discouraged; undecided whether they shall continue to have their teeth filled and refilled, or have them extracted. You examine the mouth and find eighteen or twenty teeth filled with gold in which decay has recommenced around the filling. Of course, they are not *your* fillings; they were inserted by your competitor down the street. If you are recently graduated, you say, "Fill those teeth with gold, certainly." You talk airily, indiscreetly also, about improper manipulation, and refill with gold. We are sorry for you of course, but more sorry for the patient. If, however, you have been in practice for several years, you have come to the conclusion some time past, that there are other conscientions operators in the world besides yourself, and hesitate for fear of a similar experience. "He who has not discovered for cause, has no remedy for effect." To do nothing unless you know what to do, is practice that will be pursued by every judicious practitioner. Can we diagnose the case before us? I have diagnosed it; it is a case of incompatibility of tooth structure and filling material. One of the plastic filling materials is called for. What! not use gold? Yes, in its proper place; in teeth above medium in structure nothing compares with gold as a filling material. It is as durable when properly manipulated as the rock on which the man built his house. Use gold by all means as a filling material when it is consistent with tooth preservation; but in proportion as the teeth fall below the average in density will be the number of failures with gold, though the greater the skill the better will be the result. Our only recourse, then, is a plastic filling. We must really descend and become in some degree "tooth-plasterers." It is apparent that some are prejudiced against the use of plastics, to the injury of the teeth, their owners and as often the claims of the operator. As the late Dr. Atkinson happily puts it, "Such a man ought to be prayed with."

The successful utilization of plastic fillings depends as much upon specific adaptation of means to ends, as does the successful utilization of gold depend upon manipulative ability. The plastic fillings commonly used are amalgam, cement and gutta-percha. Amalgam is the sheet anchor for the treatment of soft teeth; it answers admirably most of the requirements of a filling material, its color alone making it non-applicable to the anterior teeth. In teeth of the lowest scale of density, even amalgam has to take a second place. Nearly every one uses it nowadays more or less. No other material has had such a hard struggle for a place in dentistry. In the early days of amalgam, the better class of dentists waged war against it on "general principles"—it would lower the manipulative skill and professional standing of dentists. Time has brought its own refutation. Never in the history of dentistry has professional standing and manipulative ability been so high as to-day, notwithstanding tons of amalgam have been used as a filling material. It long ago outlived an inherited prejudice, and we are now in danger of going to the other extreme of using it when not indicated.

In filling the teeth with amalgam, the cavity should be prepared with as much care as with gold; edges should be beveled and all angles removed. When the decay is deep seated and the removal of the decomposed dentine would expose the pulp, it is excellent practice to thoroughly carbolize the cavity, cap with gutta-percha or cement, using great care to have the edges of the cavity free from decay. The enamel should be cut away until the edges become thick and strong. Always have your patient return in three or four days that you may polish the fillings with sand-paper and cuttle-fish disks. This gives the filling a smooth surface and frees it from any overhanging edges. It also adds not a little to its appearance, as it will not tarnish to the same degree as if left rough. Another consideration not to be overlooked is the opportunity to examine your work at your own leisure.

Now and then you will find a little fissure you had excavated and in your hurry had forgotten to fill. Quite as frequently you find in spite of all your warnings and the care of the patient, that while in a plastic condition the filling has been fractured. You now have an opportunity of rectifying all this. It is impossible to properly fill and polish an amalgam filling at one sitting.

A new amalgam which has received considerable attention during the past few years is composed of pure copper and mercury. I have experimented with it during the past four years, and in my estimation it is a material of considerable value. It cannot be used in places in teeth that appeal to the eye, as it discolors badly; but in soft teeth, where everything else has failed, I have found it invaluable. It is practically useful in deciduous teeth, being very plastic, and not affected by moisture as other amalgams. I know it is the fashion to condemn this material —"the blackest sheep of them all"—but it has done so well for me, in cases where I had despaired of ever putting in anything more permanent than cement, and had begun to doubt whether the fault was with the material or myself, that I must say a good word for it. One case was that of a young lady for whom I refilled with silver a dozen or more cavities that had been filled a short time before, only to find on her return for professional services six months later thirteen additional places that needed attention, this time not only cavities that had been filled by her former dentist, but also a goodly number of those I had so carefully re-treated. I refilled her teeth, using copper amalgam. A few months ago she was in my office and I had the pleasure of again examining her teeth. Two years had passed since the introduction of the copper; meanwhile she married and had a child, passing through one of the most trying periods for the teeth. Her health, if anything, was not as good as when I had last seen her, and yet I found but *three new* cavities. Comment is unnecessary.

Another case was that of a lady who had come to the conclusion that nothing could save her teeth, unless perhaps having them filled every year with cement. I found her mouth in a deplorable condition; decay had taken place around the gold and amalgam fillings; one of the worst cases I ever saw. I filled several of the worst cases with this material, the others with cement. The result was gratifying in the extreme. At the end of the year I replaced a number of the cement fillings that had worn away with the copper. She returned on a visit to her old home in New Hampshire, and while there had her teeth examined by her former dentist, who had obstinately refused to use the copper amalgam in his practice. The copper amalgam fillings in her mouth convinced him of its value. I received a letter from him some time ago, in which he stated that the amalgam in soft, frail teeth had more than exceeded his expectations. I have used the copper only in teeth far down in the scale, structurally, and am sure that I save many teeth permanently, which I could only hope to temporarily repair with any other material. I am confident that many of the failures in using this material are due to the fact that it is not mixed thoroughly. There is generally enough mercury, and to spare, in the material as it comes from the depots. It is not enough to heat the amalgam until the mercury appears—the mortar and pestal should be thoroughly warmed. Amalgam so treated is plastic in a surprisingly short time.

Cement, as a tooth preserver, stands at the head of the list. As it lasts only from one to three years, it loses much of its value; still, we occasionally find fillings of this material that have lasted longer, many times in teeth below medium in structure outwearing gold fillings. Great care should be exercised in the use of the phosphoric acid to keep it pure, as it easily absorbs water and deteriorates. The powder, if long exposed, may bring about the same result. It absorbs moisture from the air, like plaster of Paris, and becomes unfit for use. Cement requires the most careful manipulation to obtain the best results; in fact, next to gold, it requires the most skill of any material used in dentistry. I find a six-sided mixing block very handy when in a hurry, six mixing surfaces instead of the two on the ordinary block.

It is a fact that a given amount of acid only can unite with a quantity of powder sufficient to satisfy its affinity, and if there is an excess of powder, the compound formed is brittle, crumbles and admits moisture freely. Apply the rubber dam, mix the powder and acid until you have a creamy mass, trim as much as possible before it sets, and if you are particular, let your patient read the paper for thirty minutes or more, before removing the dam. Many coat the filling with sandarach or shellac varnish; but the alcoholic constituent penetrates deeper than could the saliva and does more harm than good.

Dr. Bonwill has suggested coating the filling with heated paraffine which melts at a temperature lower than wax when heated. He claims to get very good results from its use, saying that it renders the filling and interstices around it impervious to the action of acids. Dr. Flaggs suggests a mixture of one part white wax to five parts resin. This simply acts as a protective covering for twenty-four hours. I have tried Dr. Bonwill's method, but have not used it long enough to vouch for its preventing the decomposition of the oxyphosphate. Either method seems preferable to the varnishing process.

One of the most satisfactory methods of filling frail or soft teeth is found in gold inlays set with cement. The work requires time and considerable skill, and is necessarily expensive. By this means, contours can be restored, having all the appearances of gold, with the advantages of cement. For exposed positions, where appearance is an object, this method is *par excellence*. Never attempt to insert and polish an inlay at one sitting. Let the cement harden thoroughly, then make a second appointment for the final smoothing and finishing. Occasionally you find teeth which from the moment of their eruption seem markd for destruction. This is particularly true of the six-year molars, decay often commencing at a dozen different points. The surface of the enamel seems pitted in every direction. I find it easier and less expensive to the patient in the end, to immediately adjust a gold shell covering the whole tooth.

Combination fillings are quite the fad nowadays—combinations of gold and amalgam, cement and gutta-percha, etc. I have found the latter a very satisfactory method. How often we examine an oxyphosphate filling and find it to all appearances perfect, until we discover a pocket along the cervical border, leading directly to the pulp. Gutta-percha is especially valuable here. Place a small pellet at the cervical border of the cavity, finishing with cement. A chlora-percha lining is recommended for the entire cavity before filling with cement, though I have not tried it. Gutta-percha as a permanent filling has a limited field, but it is almost indispensable in cervical or buccal cavities, those exquisitively sensitive points of decay next to the gum. Where it is properly protected, I find it more valuable than gold or amalgam in low grade teeth. In using gutta-percha it is essential that the largest possible portion of both enamel and dentine should be carefully conserved. It is not important that the walls of the cavity should possess thickness or strength—every portion of enamel should be saved. Gutta-percha should always be finished from the centre toward the edges of the cavity. As a final finish, use a moderate amount of pressure to consolidate the filling.—*Odontographic Journal.*

